# The effect of ranibizumab versus photodynamic therapy on DNA damage in patients with exudative macular degeneration

**Published:** 2009-06-13

**Authors:** M Mozaffarieh, A Schötzau, T Josifova, Josef Flammer

**Affiliations:** University Eye Clinic, Basel, Switzerland

## Abstract

**Purpose:**

To compare the effect of ranibizumab treatment versus photodynamic therapy (PDT) on single-stranded DNA damage in circulating leukocytes in patients with exudative age-related macular degeneration (AMD).

**Methods:**

A comparative quantification of single-stranded DNA breaks was performed in circulating leukocytes of AMD patients before and 30 min, 45 min, 60 min, and 24 h after two different modes of therapy: a) PDT; and b) intravitreal ranibizumab injection. DNA breaks lead to smaller pieces of DNA, which in an electrical field, migrate out of the nucleus forming a tail. Damage of an individual cell was quantified as a comet tail moment. The proportion of non-zero values compared to the total number of observations was referred to as “amount of DNA damage” expressed in arbitrary units (AU). Comparisons between time points and study groups were assessed using a linear mixed-effect model.

**Results:**

PDT induced an increase in the amount of single-stranded DNA damage in the circulating leukocytes from 0.2 AU (before treatment) to 0.53 AU (30 min after treatment). This increase was significant (p=0.004). In contrast, after ranibizumab treatment, the DNA damage in the circulating leukocytes remained unchanged.

**Conclusions:**

PDT purposely induces a local oxidative stress to damage the newly formed vessels. Our results indicate an additional systemic oxidative stress, apparent as amount of single-stranded DNA damage in the circulating leukocytes, for at least 30 min after treatment.

## Introduction

Age-related macular degeneration (AMD) is a leading cause of irreversible blindness [1,2]. The overall prevalence of advanced AMD is projected to increase by about 50% by the year 2020 [3]. One important factor in the pathogenesis is oxidative stress [4-6], which is molecular damage (including DNA) by reactive oxygen species [7]. DNA damage can occur as double-strand breaks, which result from damages in opposite strands of the DNA helix, or as single–strand breaks, which result when only one of the two strands of a double helix has a defect [8]. The amount of DNA damage in the human body depends on cell type, cell age, patient DNA age, repair capacity, and on exogenous factors such as oxidative stress [9-11]. Increased DNA damage has been demonstrated in other ocular pathologies, such as glaucoma, where oxidative damage also plays a role [12,13].

Photodynamic therapy (PDT) was a common therapy for exudative AMD until it was replaced largely by intravitreal application of vascular endothelial growth factor (VEGF) inhibitors such as ranibizumab [14]. In PDT, a light-sensitive dye, verteporfin, is injected intravenously. As it is bound to low density lipoprotein (LDL), it binds predominantly to cells with high metabolic activity such as endothelial cells of newly formed vessels. The pathological tissue is purposely damaged with laser light, exciting the photosensitizer. The photosensitizer transfers energy to a neighboring oxygen molecule, turning it into singlet oxygen, which induces oxidative damage to newly formed vessels.

Using PDT we purposely induced local oxidative stress. We tested the hypothesis of an additional systemic oxidative stress as a side effect of treatment. We therefore compared the effect of PDT versus ranibizumab treatment on the amount of single-stranded DNA damage in circulating leukocytes.

## Methods

### Study design

Patients with exudative AMD were recruited from the University Eye Clinic Basel between January 2006 and September 2007. Ethical approval was obtained from the local medical ethics committee, and written informed consent was received from all participants before entry into the study. The study was designed and conducted in accordance with the tenets of Declaration of Helsinki, and 12 patients were recruited.

All patients received a standard ophthalmic examination, including visual acuity measurement, slit-lamp biomicroscopy, and dilated fundus examination that was performed by a retinal specialist. The diagnosis of exudative AMD was based on ophthalmoscopic and fluorescein angiographic findings. Inclusion criteria for patients were as follows: 1) age of 50 years or older; 2) classic subfoveal choroidal neovascularization (CNV) on fluorescein angiography in one eye; 3) first-time treatment of PDT. Exclusion criteria included the following: 1) history of other ocular or systemic disease (e.g., diabetes mellitus), smoking, drug or alcohol abuse, trauma, infection, or inflammation; 2) macular lesions associated with other eye diseases, such as degenerative myopia, angioid streaks, or any other retinal/choroidal diseases.

### Study treatment

After enrollment in the study, patients with a subfoveal classic CNV were randomly selected by our vitreoretinal specialist (T.J.) to receive either verteporfin PDT or an intravitreal injection of 0.5 mg of ranimizumab. Only one eye per patient was chosen as the study eye, and only the study eye received treatment. If both eyes were eligible, the eye with the better visual acuity was selected for treatment. In addition, 20 ml blood samples were obtained by venipuncture from all patients both before treatment and 30 min, 45 min, 60 min, and 24 h after treatment for analysis of DNA damage by comet assay. Table 1 depicts the demographic data of the two groups of patients.

**Table 1:** Demographic data of the patients

**Type of treatment**	**PDT**	**Ranibizumab**	**p value**
N	6	6	
Age	66 (10.5)	67 (10.2)	n.s.*
Male	50%	33%	n.s.**
Female	50%	67%	n.s.**

Age is expressed as mean and standard deviation (SD). The asterisk represents *t*-test, and the double asterisk represents Fisher’s exact test, and n.s. represents not significant. There were no significant differences in age or gender between the group of patients treated with PDT and the one treated with ranibizumab.

#### PDT

Verteporfin (Dose: 6 mg/m^2^ body surface area ; Infusion rate: 3 ml/min) was infused through intravenous access over a 10 min period. Approximately 15 min after the start of the infusion, laser light of 689 nm was applied for 83 s to the CNV lesion through a fundus contact lens.

#### Intravitreal ranibizumab injection

Under topical anesthesia (tetracaine 1% eye drops) and sterile conditions, 0.5 mg of ranibizumab was injected with a 30 gauge needle inserted 3.5 to 4 mm posterior to the limbus through the sclera into the vitreous cavity behind the lens of the eye.

### Comet assay analysis

#### Isolation of leukocytes

Blood samples (20 ml) were obtained by venipuncture from the two groups of patients and collected in heparinized tubes. The leukocytes were isolated using Ficoll-Histopaque gradients (Histopaque 1077; Sigma-Aldrich, Zurich, Switzerland) [12]. Two ml of histopaque are placed into 10 ml sterile centrifuge tubes and 5 ml of diluted blood samples are carefully layered onto each histopaque gradient. Gradients are centrifuged at 800x g for 15 min. The leukocyte bands were removed from the interface between plasma and the histopaque layers of each tube and collected into one 50 ml tube. The total volume was brought to 50 ml with cold Dulbecco’s Modified Eagle Medium (DMEM; Gibco, Invitrogen, Basel, Switzerland). The cell suspension was washed three times with DMEM, and the total number of cells was determined. Cells were finally suspended in phosphate buffered saline (PBS 1X; 0.14 M NaCl, 0.003 M KCl, 0.002 M K_2_HPO_4_, 0.01M Na_2_HPO_4_) and aliquoted into Eppendorf tubes at 10^5^ cells/tube. After centrifugation at 250x g for 10 min, cell pellets were stored at −80 °C.

#### Gel electrophoresis

**Figure 1:**
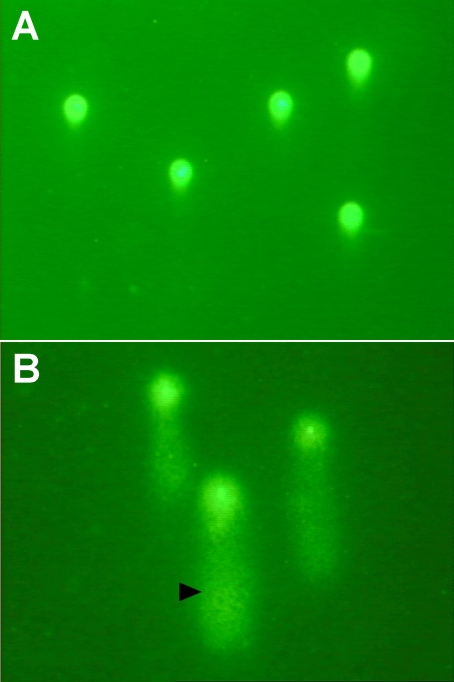
Photographs of cells analyzed by comet assay analysis. **A:** Photograph **A** depicts intact cells (without tail) of a patient treated with ranimizumab. **B:** Photograph **B** depicts cells of a patient 30 min after treatment with PDT. Arrowhead points to a typical “comet” with a bright head and tail.

Gel electrophoresis separates damaged DNA from undamaged DNA. This method has previously been described in detail in the literature [15]. The cells under study were embedded in agarose on a slide and subjected to lysis followed by electrophoresis under specific conditions. DNA is negatively charged, in particular, in alkaline conditions. When put in an electrical field, the intact DNA was such a large molecule that it hardly moved. DNA breaks, however, lead to smaller pieces of DNA which migrated away from the intact DNA. The amount of migrated DNA was the measure of the extent of DNA damage. To detect DNA, the slides were stained with cyber-green and examined by fluorescence microscopy equipped with a personal computer based analysis system (Kinetic Imaging; Nikon, Zürich, Switzerland), which enabled quantification of DNA damage. Cells containing damaged DNA had the appearance of a comet with a bright head (undamaged) and tail (damaged; Figure 1).

#### Quantification of DNA damage

It is recommended by manufacturers (Trevigen, Zürich, Switzerland) that 50 cells on each slide be chosen at random for quantification of DNA damage using the computer software. The tail moment is defined as the product of the tail length and the fraction of total DNA in the tail:

tail moment=tail length × % of DNA in the tail

This is calculated automatically by the comet assay computer software system (Nikon) as an average for the 50 cells selected for measurement.

### Statistical analysis

The main parameter for the statistical evaluation was the “tail moment.” As the parameter was zero-inflated (had many zeros), its distribution was heavy-tailed. Therefore, the assumption for regression modeling was violated. To overcome this problem, we calculated the proportion of nonzero values compared to the total number of observations for each blood sample of each participant. These proportions were fairly good “log normal” distributed. To simplify matters, we called these proportions the “amount of DNA damage.” Comparisons between time points and study groups were assessed using a linear mixed-effect model as will be described.

**Figure 2:**
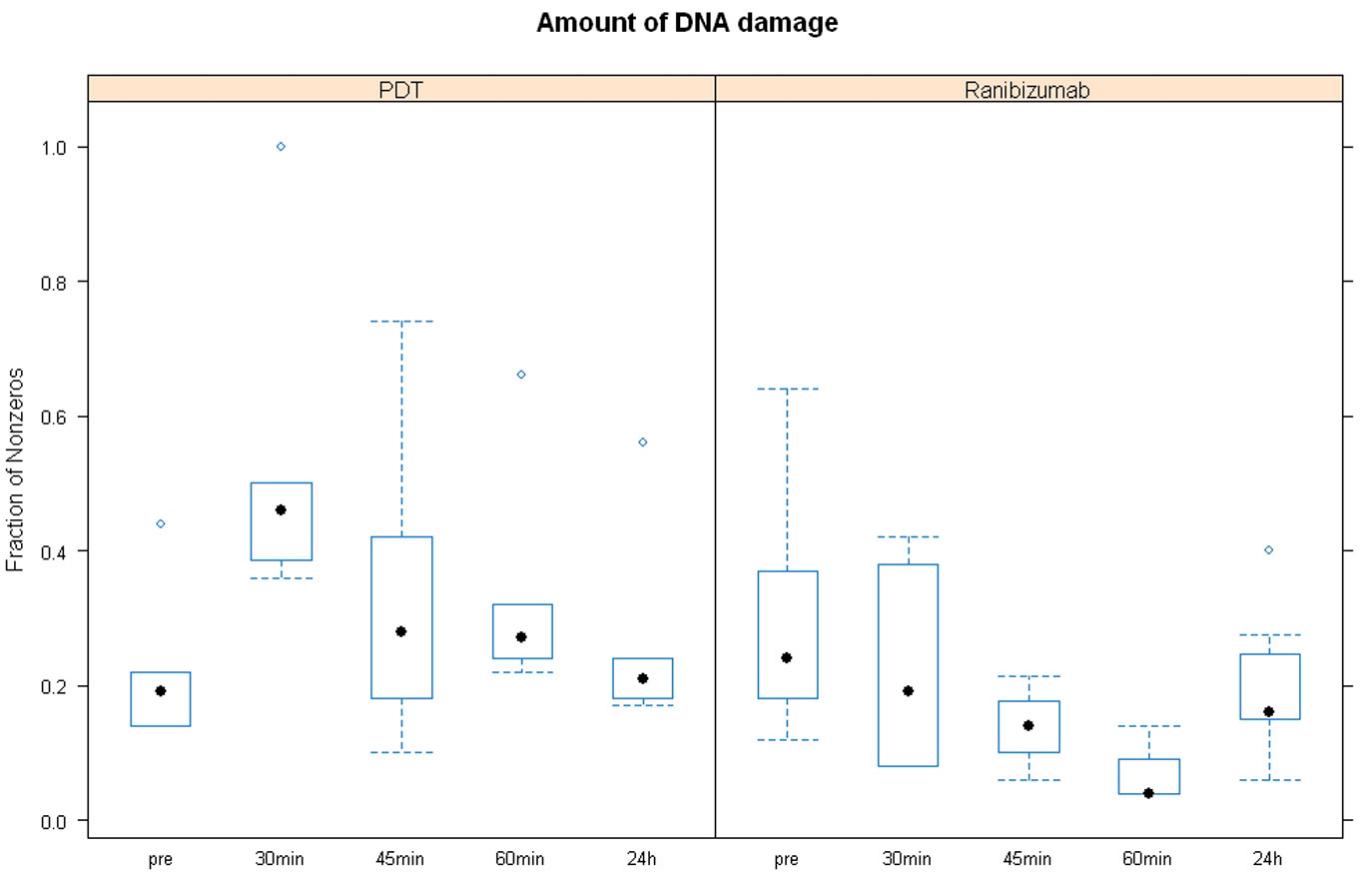
Amount of DNA damage before and after PDT and ranibizumab treatment. With the exception of columns marked pre, all times are post treatment. PDT induced an increase in the amount of single stranded DNA damage in the circulating leukocytes from 0.22AU (before treatment) to 0.53 AU (30 min after treatment).

To explore the effect of time for PDT and ranibizumab, we performed a linear mixed-effect model with fixed factor “time” and random factor “subject” on the log-transformed proportions. This model allowed comparisons before treatment with postoperative treatment at the various times of 30 min, 45 min, 60 min, and 24 h. Descriptive statistics and corresponding box plots are reported in Table 2 and Figure 2. A p-value <0.05 was considered significant. The p-values of the statistical tests were interpreted in a purely exploratory manner and were not adjusted for multiple comparisons. All analyses were done using the statistical software R, version 7.1.

**Table 2:** Descriptive statistics for the amount of DNA damage before and after PDT and ranibizumab treatment

**Type of treatment**	**Photodynamic therapy (PDT)**					**Ranibizumab**				
	**Prior to treatment**	**30 min**	**45 min**	**60 min**	**24 h**	**Prior to treatment**	**30 min**	**45 min**	**60 min**	**24 h**
Mean	0.22	0.53	0.33	0.23	0.20	0.30	0.22	0.14	0.13	0.15
Median	0.19	0.46	0.28	0.21	0.19	0.24	0.19	0.14	0.16	0.16
StdDev	0.11	0.24	0.23	0.12	0.11	0.18	0.16	0.08	0.06	0.08
Minimum	0.14	0.36	0.10	0.13	0.13	0.12	0.08	0.06	0.06	0.06
Maximum	0.44	1.00	0.74	0.46	0.45	0.64	0.42	0.21	0.20	0.21
N	6	6	6	6	6	6	6	6	6	6
p value		p=0.004*	ns	ns	ns	ns	ns	ns	ns	ns

PDT induced a transient increase in the amount of single stranded DNA damage in the circulating leukocytes from 0.22 AU (before treatment) to 0.53 AU (30 min after treatment).The asterisk indicates a significant result was only obtained after 30 min.

## Results

### Amount of DNA damage

Patients treated with PDT (n=6) showed a significant increase in the amount of DNA damage in the circulating leukocytes from 0.2 AU (before treatment) to 0.53 AU (30 min after treatment) (p=0.004; Figure 2). At all other postoperative times no significant differences in the amount of comet tails in comparison to before treatment were accounted for. In the ranibizumab-treated group (n=6), however, there was a slight, yet not significant tendency for a decrease in the DNA damage up to 45 min (see Table 2).

## Discussion

In this study we quantified the amount of DNA damage in circulating leukocytes of patients with exudative AMD before and 30 min, 45 min, 60 min, and 24 h after either PDT or intravitreal ranibizumab injection. There was a significant increase in DNA damage 30 min after treatment with PDT. No such increase in DNA damage was observed after intravitreal ranibizumab treatment.

DNA damage can result from a variety of factors including ultraviolet light, X-rays, ionizing radiation, toxins, chemicals, or reactive oxygen species [16-22]. The most likely reason for a higher rate of DNA damage shortly after PDT treatment is oxidative stress induced by the illumination of a photosensitizer. Photosensitizers utilize energy from light to turn ground state oxygen (O_2_; the most stable state of oxygen) into reactive oxygen species [23,24]. In the ground state, the last two electrons of the oxygen molecule are located in a different p* antibonding orbital. These two unpaired electrons have the same quantum spin number (they have parallel spins). If ground state oxygen absorbs sufficient energy to reverse the spin of one of its unpaired electrons, the two unpaired electrons then have opposite spins. This activated form of oxygen is called singlet oxygen (1O_2_) [25]. Singlet oxygen is much more reactive than ground state oxygen and disrupts CNV [26,27]. Therefore, the neovascular lesions are ultimately destroyed by an iatrogenic source of oxidative stress. Paradoxically, oxidative stress, which plays a key role in the pathogenesis of AMD [28], is used as a treatment modality [14], and this, in turn, leads to a short-term systemic oxidative stress.

The systemic oxidative stress observed 30 min after PDT was short-term and transient in nature, rather than long-term. Injury to DNA is minimized by systems that recognize and correct the damage [29]. DNA breaks are therefore the result of balance between the damaging events occurring in DNA and the repair mechanisms reversing the damage to DNA [30]. In our investigation we quantified single–stranded DNA damage. This type of damage [31,32] is repaired at a faster rate than double-stranded DNA damage. As PDT is used in several medical fields, such as oncology [33,34], dermatology [35-37] or cosmetic surgery [38], knowledge of a transient systemic oxidative stress may eventually lead to the use of systemic antioxidative treatment in combination with PDT.

The study design did not allow a differentiation as to how much of the oxidative stress expressed as DNA damage was due to laser radiation of the macula and how much was due to environmental light reaching both the eye and the skin. It would have been interesting to compare our results of systemic oxidative stress in patients with exudative AMD to those with dry AMD. Further investigations are needed.

Treatment with PDT requires intravenous administration of verteporfin with maximum systemic exposure immediately after perfusion. However, ranibizumab is administrated by intravitreal injection, and systemic exposure after intravitreal administration of a drug is dependent of its pharmacokinetics. Ranibizumab is distributed rapidly to the retina after 6 to 24 h [39]. Moreover, serum concentrations of ranibizumab were shown to be very low [39], reflecting wider distribution and faster clearance when the drug reached the serum. Our results support this investigation. Our quantitative analysis of DNA breaks after 1 h and 24 h after treatment were not significantly different from each other, showing that there was no significant systemic oxidative stress after ranibizumab treatment. But even if serum concentrations of ranibizumab were higher, we would still not expect an increase in systemic oxidative stress after treatment with a recombinant, humanized monocloncal Fab fragment that neutralizes all active forms of VEGF- A [40]. Ranibizumab is thus not only more effective [41], it probably also induces less systemic side effects. In conclusion, PDT may lead to a transient systemic oxidative stress as observed by a higher rate of DNA damage after PDT treatment. Ranibizumab has no such side effects.
